# Targeting WEE1 to Overcome *ARID1A* Mutation-Driven Osimertinib Resistance in *EGFR*-Mutant Lung Cancer

**DOI:** 10.1016/j.jtho.2025.06.007

**Published:** 2025-06-13

**Authors:** Koji Fukuda, Shigeki Nanjo, Shinji Takeuchi, Turja Chakrabarti, Tyiesha Brown, Sharon Wesley Dev Sahadevan, Sachiko Arai, Shigeki Sato, Hiroshi Kotani, Akihiro Nishiyama, Hiroyuki Sakaguchi, Koushiro Ohtsubo, Hiroaki Taniguchi, Collin M. Blakely, Trever G. Bivona, Seiji Yano

**Affiliations:** a Division of Innovative Cancer Control Research, Cancer Research Institute, Kanazawa University, Kanazawa, Japan; b Nano Life Science Institute, Kanazawa University, Kanazawa, Japan; c Department of Respiratory Medicine, Faculty of Medicine, Institute of Medical, Pharmaceutical, and Health Sciences, Kanazawa University, Kanazawa, Japan; d Department of Medical Oncology, Kanazawa University Hospital, Kanazawa, Japan; e Department of Medicine, University of California, San Francisco, California; f Chan-Zuckerberg, Biohub, San Francisco, California

**Keywords:** Osimertinib, *ARID1A* mutation, WEE1 kinase, Leptomeningeal carcinomatosis, Drug resistance

## Abstract

**Introduction:**

Osimertinib, a third-generation EGFR tyrosine kinase inhibitor (EGFR-TKI), is commonly used as a first-line treatment for *EGFR*-mutant NSCLC. Nevertheless, despite its efficacy, resistance remains a major clinical challenge with unknown underlying mechanisms. This study aimed to investigate the mechanisms driving osimertinib resistance and identify therapeutic strategies.

**Methods:**

Using a mouse model of leptomeningeal carcinomatosis, we induced osimertinib resistance and performed next-generation sequencing to characterize resistance-associated mutations. We also analyzed clinical samples to correlate *ARID1A* status with progression-free survival and overall survival in patients receiving osimertinib.

**Results:**

Mutations in the AT-rich interacting domain-containing protein 1A (*ARID1A*) gene were the most prevalent in resistant cells. Functional assays revealed that *ARID1A* knockout in parental cells and wild-type *ARID1A* gene expression in resistant cells were critical in conferring osimertinib resistance. A Clustered Regularly Interspaced Short Palindromic Repeats-Cas9 knockout screen identified WEE1 kinase as a potent enhancer of apoptosis in *ARID1A*-mutant osimertinib-resistant cells. Mechanistically, *ARID1A*-mutant cells exhibited reduced expression of genes involved in cell cycle regulation and DNA repair, rendering them particularly sensitive to WEE1 inhibition. In the leptomeningeal carcinomatosis mouse model, the combined inhibition of EGFR and WEE1 significantly suppressed tumor growth. Clinically, patients with *ARID1A* mutations treated with osimertinib had significantly shorter median progression-free survival (6.25 versus 18.0 months, *p* = 0.0036) and overall survival (17.0 versus 34.0 months, *p* = 0.024) than did those with wild-type *ARID1A*.

**Conclusions:**

These findings suggest that *ARID1A* mutations are critical biomarkers for osimertinib resistance and highlight WEE1 inhibition as a promising therapeutic approach for *ARID1A*-mutant osimertinib-resistant NSCLC.

## Introduction

In the EGFR, activating mutations have been reported in approximately 10% of Caucasian and 50% of East Asian patients with NSCLC.^1^ Central nervous system (CNS) metastases have also been observed in 15% to 30% of patients treated with EGFR tyrosine kinase inhibitors (EGFR-TKIs), with an even higher prevalence in those harboring *EGFR* mutations than in those without them.^2^ Leptomeningeal carcinomatosis (LMC), an aggressive form of CNS metastasis, is observed in 3.4% to 3.8% of patients with NSCLC, with an incidence of 9.4% among those with *EGFR* mutations.^3^ LMC remains challenging owing to rapid progression and limited drug penetration across the blood-brain barrier. This necessitates effective control of CNS metastases to successfully manage *EGFR*-mutant NSCLC.

First-generation EGFR-TKIs, such as gefitinib and erlotinib,^3,4^ have revealed remarkable efficacy in treating *EGFR*-mutant NSCLC. Nevertheless, recurrence in the CNS remains particularly challenging. Osimertinib, a third-generation EGFR-TKI, selectively targets activating *EGFR* mutants and the resistance-associated *EGFR*-T790M mutation while sparing wild-type *EGFR* and other kinases.^5^ The FLAURA trial established osimertinib as the standard first-line treatment for *EGFR*-mutant NSCLC, finding superior progression-free survival (PFS)^6^ to, and significantly improved overall survival (OS)^7^ compared with, gefitinib and erlotinib.

Although osimertinib has reported decent efficacy against CNS metastases, with an objective response rate of 64% and a disease control rate of 90%,^8^ resistance remains a significant clinical problem. Known resistance mechanisms include acquisition of the *EGFR*-C797S mutation,^9^ loss of the T790M mutation, activation of bypass pathways (e.g., MET, HER2, and KRAS), and histologically diagnosed transformations, such as small-cell transformation^5^ and epithelial-mesenchymal transition.^10,11^ Nevertheless, in a substantial proportion of patients (40%–50% in first-line therapy and 30%–40% in second-line therapy), resistance develops through unknown or poorly understood mechanisms.^12^ Unraveling these mechanisms is critical for improving outcomes in patients with *EGFR*-mutant NSCLC. Therefore, this study aimed to explore the mechanisms involved in osimertinib resistance and identify potential therapeutic strategies.

## Materials and Methods

The complete materials and methods of this study can be found in the Supplementary Material.

### Cell Lines

PC9 (RRID: CVCL_B260, EGFR exon 19 deletion, NSCLC) was purchased from the RIKEN Cell Bank. The H1975 (RRID: CVCL_1511, EGFR L858R/T790M), NCI-H2279 (RRID: CVCL_9642, EGFR exon 19 deletion), and H2935 (EGFR L858R) human lung adenocarcinoma cell lines were provided by Dr. Yoshitaka Sekido (Aichi Cancer Center Research Institute). All cells were maintained in RPMI-1640 medium supplemented with 10% fetal bovine serum, penicillin (100 U/mL), and streptomycin (10 *μ*g/mL) in a humidified CO_2_ incubator at 37 °C. Cells were passaged for less than 3 months before being renewed from frozen, early-passage stocks.

The cells were monthly screened for mycoplasma using the MycoAlert Mycoplasma Detection Kit (Lonza). Cell line authentication was performed using short tandem repeat analysis at the National Institute of Biomedical Innovation (Osaka, Japan).

## Results

### Detection of ARID1A Mutation in PC9 Osimertinib-Resistant Cells

**Figure 1.**
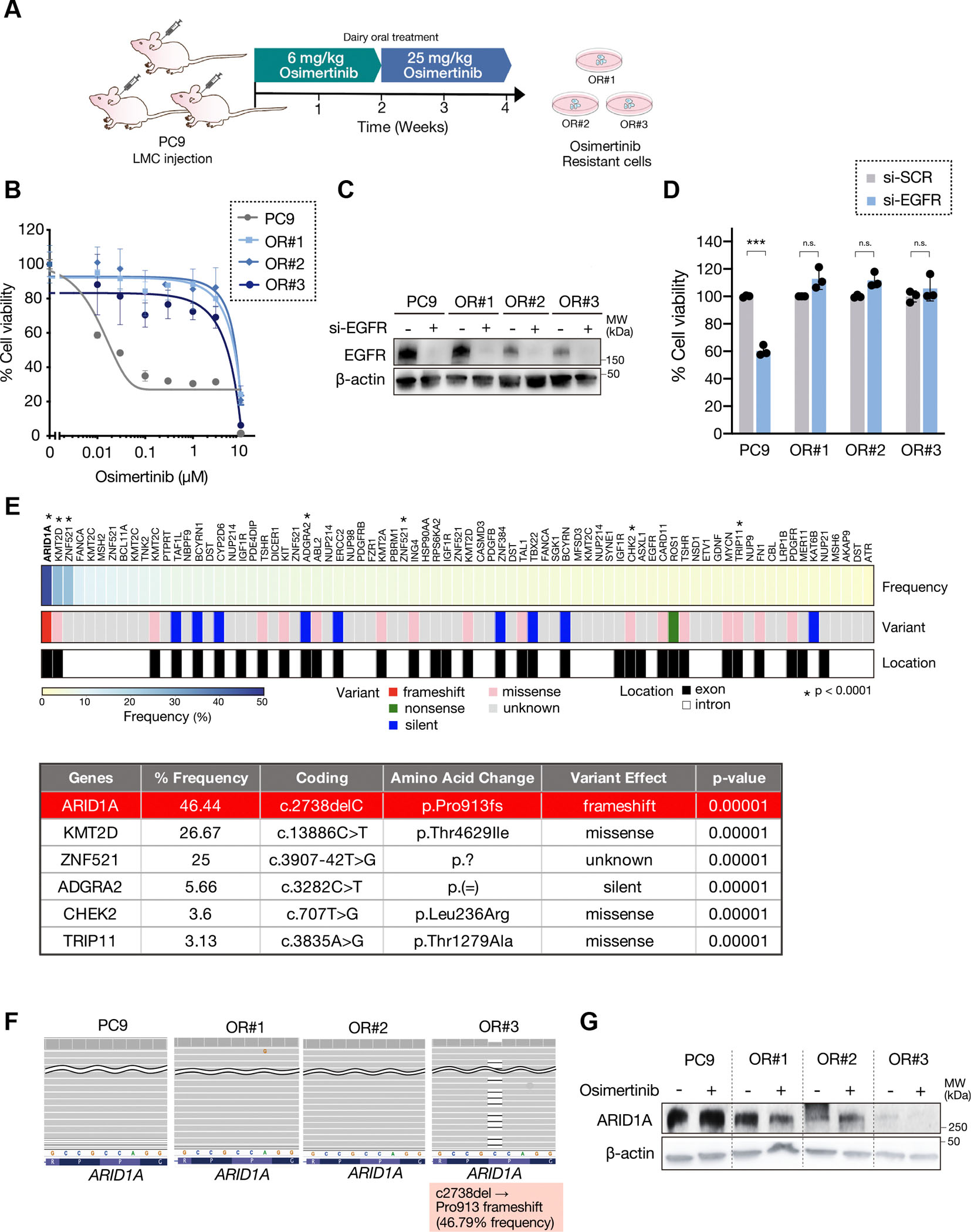
Detection of *ARID1A* mutation in PC9 osimertinib-resistant cells. Schematic illustration indicating the establishment of osimertinib-resistant cell lines. (*B*) PC9 parental cells and three resistant cell lines (OR#1, OR#2, and OR#3) were treated with various concentrations of osimertinib, and cell viability was measured using 3-(4,5-dimethylthiazol-2-yl)-2,5-diphenyltetrazolium bromide (MTT) assay after 72 hours. Data are presented as the mean ± SD of triplicate experiments. (*C*) PC9 parental and resistant cell lines were transfected with small interfering RNAs targeting *EGFR* for 48 hours. Cell lysates were analyzed using Western Blotting with the indicated antibodies. (*D*) Cell viability after small interfering RNA transfection was assessed using the MTT assay at 72 hours. Data are reported as mean ± SD of triplicate experiments. Statistical significance was determined using the Student’s *t* test (***p < 0.001). (*E*) Genomic DNA from OR#3 cells was analyzed for mutations using the Ion AmpliSeq Comprehensive Cancer Panel, which covers 409 cancer-associated genes. Mutation frequency, variant type, and location data are displayed. Statistical significance was determined using the Student’s *t* test (*p = 0.00001). (*F*)A comparison of genomic DNA from PC9 parental and resistant cell lines identified the *ARID1A* c2738del mutation exclusively in OR#3 cells. (*G*) Cell lysates from PC9 parental and resistant lines treated with 1*μ*M osimertinib for 48 hours were analyzed by Western Blotting using the indicated antibodies. si-EGFR, EGFR-specific small interfering RNA; si-SCR, scrambled control small interfering RNA. Panels *B* to *D* were adapted with permission from Cancer Science, 2021;112:3784–3795, Wiley.^13^

We have previously developed osimertinib-resistant cell lines derived from three different mouse LMC models,^13^ namely, PC9-OR#1, PC9-OR#2, and PC9-OR#3, by subjecting cells to continuous osimertinib treatment for 1 month (Fig. 1A). All three resistant cell lines exhibited a greater than 200-fold increase in resistance to osimertinib than did parental PC9 cells (Fig. 1B). Notably, none of the resistant cell lines carried the *EGFR*-C797S mutation, and all retained the *EGFR* exon-19 deletion found in the parental PC9 cells. Furthermore, *EGFR* knockdown using specific small interfering RNAs (siRNAs) significantly reduced the viability of parental PC9 cells, although it had no impact on the resistant cell lines (Fig. 1C and D), suggesting that resistance in these cells operates independently of EGFR signaling.

To identify potential genetic alterations underlying resistance, we extracted genomic DNA and screened for mutations or amplifications across 409 cancer-associated genes using the Ion AmpliSeq Comprehensive Cancer Panel. Notably, an *ARID1A* mutation (Pro913fs) was detected exclusively in PC9-OR#3 cells but not in parental PC9 cells or the two remaining resistant lines, PC9-OR#1 and PC9-OR#2 (Fig. 1E and F). Consistent with this result, ARID1A expression was markedly reduced in PC9-OR#3 cells compared with that in parental cells and other resistant lines (Fig. 1G). No additional known mutations or amplifications associated with osimertinib resistance were identified in PC9-OR#3 cells, indicating that the *ARID1A* mutation may play a central role in resistance development.

To further evaluate the role of ARID1A in osimertinib resistance, we established four clonal cell lines derived from PC9-OR#3 cells through limiting dilution. All four clones harbored the *ARID1A*-Pro913fs mutation in the parental PC9-OR#3 cells and exhibited similar levels of resistance to osimertinib (Supplementary Fig. 1A and B). The *ARID1A* mutation introduced a frameshift, causing a premature termination codon (Supplementary Fig. 1C). This mutation likely leads to the production of an incomplete protein that undergoes proteasomal degradation. Supporting this hypothesis, we observed reduced ARID1A expression across all four clonal cell lines (Supplementary Fig. 1D).

These findings indicate that the *ARID1A*-Pro913fs mutation and the associated reduction in ARID1A expression are pivotal in osimertinib resistance in PC9-OR#3 cells.

### ARID1A Deletion Induced Osimertinib Resistance in EGFR-Mutant NSCLC Cells

**Figure 2.**
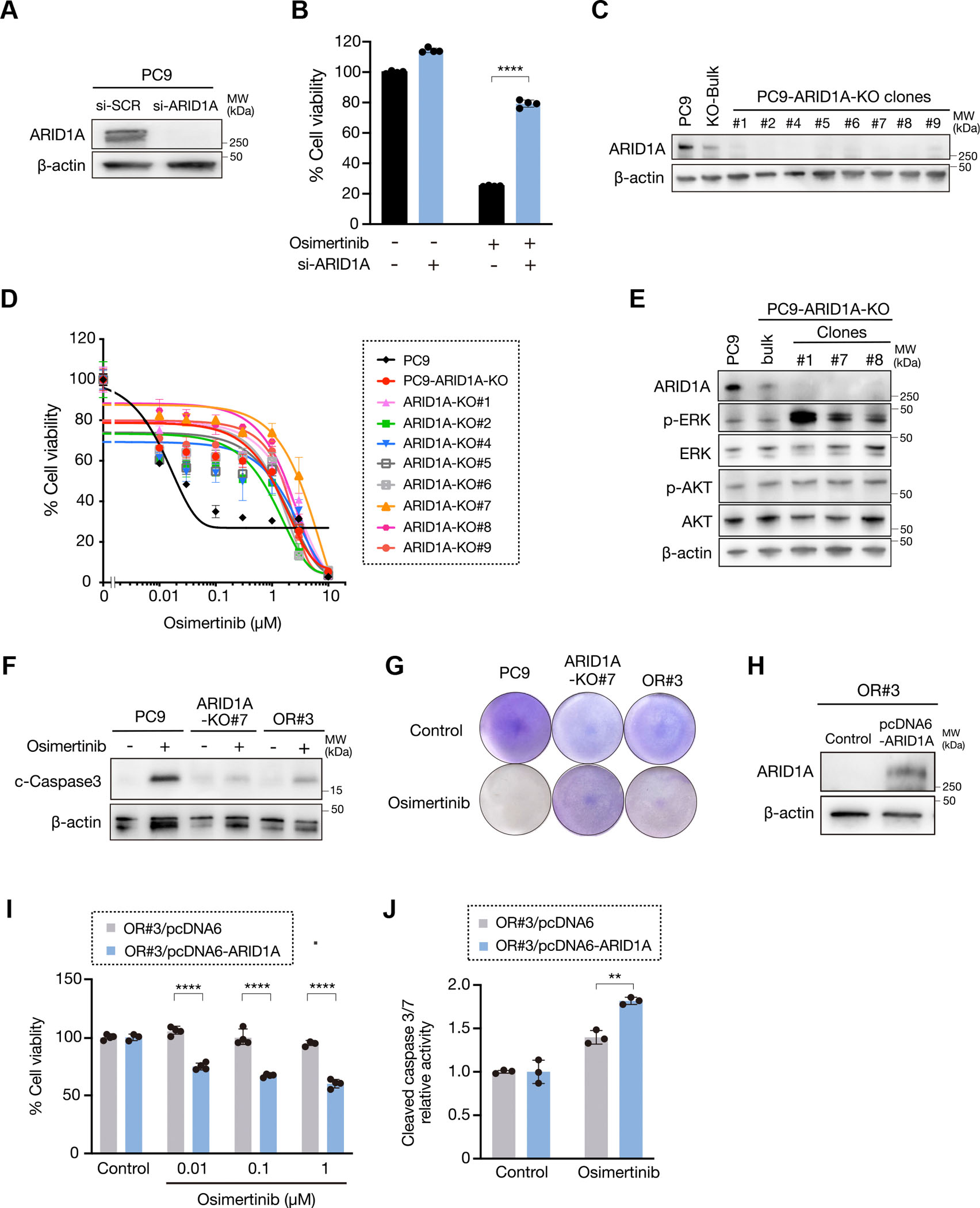
ARID1A deletion induces osimertinib resistance in EGFR-mutant NSCLC cells. (*A*) PC9 cells were transfected with siRNAs targeting ARID1A for 48 hours. Cell lysates were analyzed by Western Blotting with the indicated antibodies. (*B*) PC9 cells were transfected with small interfering RNAs targeting ARID1A and treated with 1 *μ*M osimertinib. Cell viability was measured using the MTT assay at 72 hours. Bars represent the mean ± SD of triplicate experiments. Statistical significance was determined using the Student’s *t* test (****p < 0.0001). (*C)* Cell lysates from PC9, PC9 ARID1A KO bulk cells (PC9-ARID1A-KO-Bulk), and clonal cell lines were analyzed using Western Blotting with the indicated antibodies. (*D*) PC9, PC9-ARID1A-KO-Bulk, and clonal cell lines were treated with osimertinib at various concentrations for 72 hours. Cell viability was measured, and data are presented as the mean ± SD of triplicate experiments. (*E*) Western Blot analysis of cell lysates from PC9, PC9-ARID1A-KO-Bulk, and clonal cell lines (#1, #7, and #8) using the indicated antibodies. (*F*) PC9, PC9-ARID1A-KO#7, and OR#3 cells were treated with 1 *μ*M osimertinib for 72 hours. Cell lysates were analyzed using Western Blotting with the indicated antibodies. (*G*) Cell growth after 7 days of treatment with 1 *μ*M osimertinib was assessed using crystal violet staining. (*H*) OR#3 cells were transfected with pcDNA6-ARID1A, and cell lysates were analyzed using Western Blotting with the indicated antibodies. (*I*) OR#3/pcDNA6-ARID1A cells were treated with osimertinib at various concentrations for 72 hours. Cell viability was measured using the MTT assay. Bars represent the mean ± SD of triplicate experiments. Statistical significance was determined using Student’s *t* test (****p < 0.0001). (*J*) Apoptosis was quantified using the Caspase-Glo 3/7 assay. Bars represent the mean ± SD of triplicate experiments. Statistical significance was determined using Student’s *t* test (**p < 0.01). si-SCR, scrambled control small interfering RNA.

To determine whether *ARID1A* mutations directly contribute to osimertinib resistance, we knocked down ARID1A expression in PC9 cells using *ARID1A*-specific siRNA (Fig. 2A). ARID1A knockdown significantly decreased the sensitivity of PC9 cells to osimertinib (Fig. 2B), consistent across three additional *EGFR*-mutant cell lines—H2279, H2935, and H1975—where ARID1A knockdown similarly induced osimertinib resistance (Supplementary Fig. 2A and B). To validate these findings, we used the Clustered Regularly Interspaced Short Palindromic Repeats (CRISPR)-Cas9 system to generate *ARID1A*-knockout (ARID1A-KO) cells. After establishing PC9-ARID1A-KO-Bulk cells, clonal cell lines were isolated using flow cytometry. Western blot analysis confirmed a significant reduction in ARID1A expression across all clonal lines (Fig. 2C). Importantly, eight of these ARID1A-KO clones exhibited an increase in resistance to osimertinib that was greater than 200-fold that of parental PC9 cells (Fig. 2D). Next, we analyzed downstream signaling pathways in the three most osimertinib-resistant clones (KO#1, KO#7, and KO#8). Phosphorylation of ERK was consistently elevated in all three clones; nevertheless, AKT phosphorylation did not increase (Fig. 2E). Similarly, in PC9-OR#3 cells, ERK phosphorylation was strongly elevated, whereas AKT phosphorylation was reduced (Supplementary Fig. 3A). Co-treatment with the MEK inhibitor trametinib and osimertinib reduced cell viability in ARID1A-KO#7 and PC9-OR#3 cells but failed to fully suppress proliferation to the level achieved by osimertinib monotherapy in parental PC9 cells (Supplementary Fig. 3B). These findings suggest that ARID1A deletion partially contributes to resistance through MAPK signaling activation.

The impact of ARID1A deletion on apoptosis was also evaluated. Treatment with osimertinib induced cleaved caspase activity in parental PC9 cells but not in ARID1A-KO#7 or PC9-OR#3 cells (Fig. 2F). These findings were further corroborated by a cell growth assay conducted after an additional week of treatment, which found sustained growth in ARID1A-KO cells despite osimertinib exposure (Fig. 2G). To determine whether ARID1A re-expression could reverse osimertinib resistance, ARID1A was introduced into PC9-OR#3 cells using the pc-DNA6-ARID1A plasmid (Fig. 2H). ARID1A re-expression significantly reduced osimertinib resistance in PC9-OR#3 cells (Fig. 2I) and restored cleaved caspase-3 activity (Fig. 2J). These results indicate that reduced ARID1A expression drives osimertinib resistance in *EGFR*-mutant NSCLC.

### CRISPR/Cas9 KO screening identified WEE1 inhibition as a potential therapeutic target in *ARID1A*-mutant resistant cells

**Figure 3.**
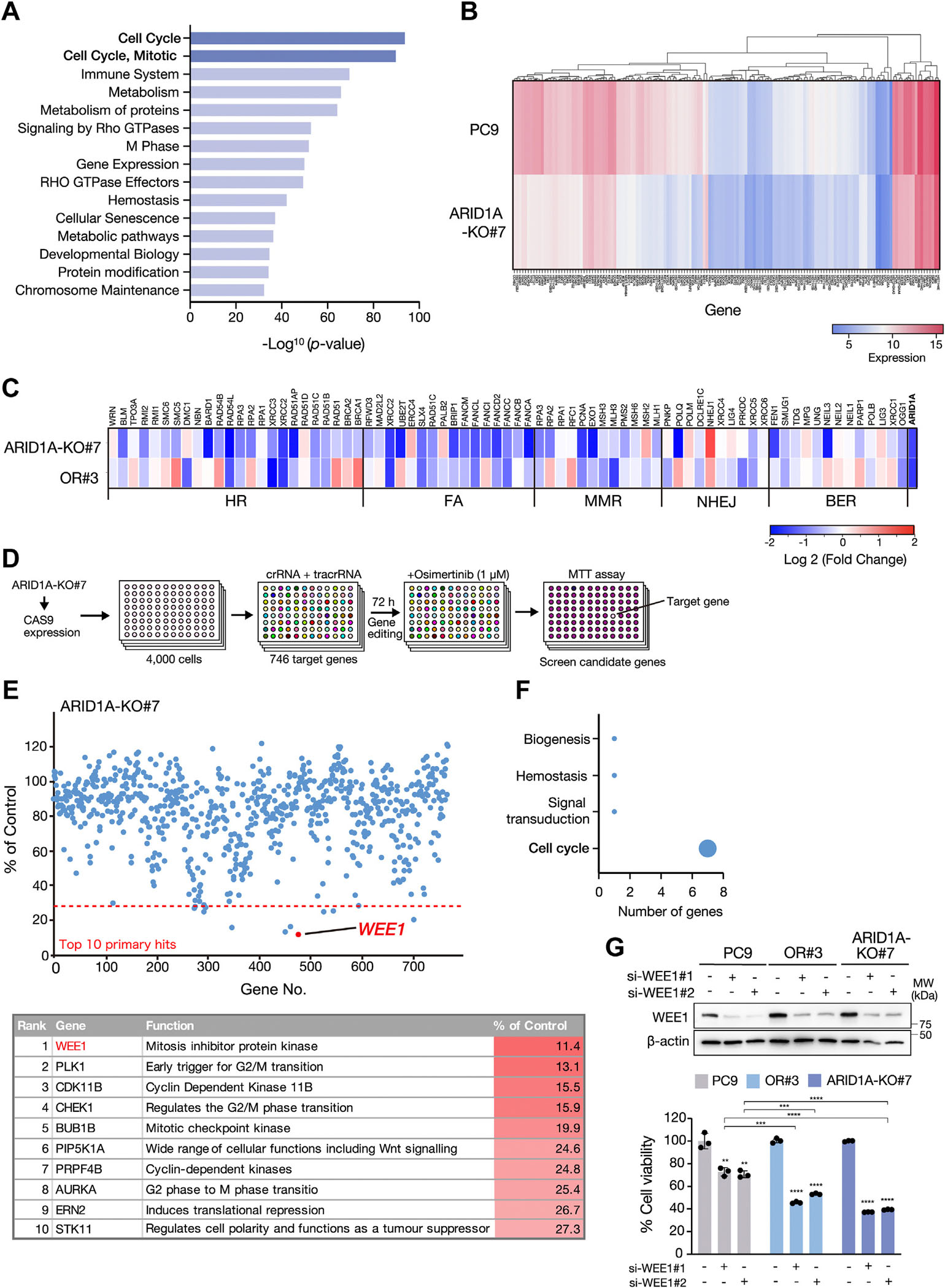
Clustered Regularly Interspaced Short Palindromic Repeats (CRISPR)/Cas9 KO screening identifies WEE1 inhibition as a promising therapeutic target in *ARID1A*-mutant resistant cells. (*A*) Comprehensive gene expression and pathway analyses comparing PC9 parental cells and ARID1A-KO#7 cells. (*B*) Heatmap depicting differential gene expression of PC9 and ARID1A-KO#7 cells. (*C*) Heatmap illustrating gene expression changes within the DNA damage response pathway in PC9 and ARID1A-KO#7 cells. (*D*) Schematic representation of the functional genomic CRISPR-KO screening process. (*E*) ARID1A-KO#7 cells expressing Cas9 were treated with a CRISPR RNA library for 7 days. Cell viability was assessed using the MTT assay at 72 hours. The top 10 primary hits and corresponding cell viability results are listed in the table, bottom left. (*F*) Pathway analysis of the top 10 genes identified from the CRISPR-KO screening, highlighting their roles across various biological pathways. (*G*) PC9 parental cells, OR#3 cells, and ARID1A-KO#7 cells were transfected with small interfering RNAs targeting WEE1. Cell lysates were collected after 48 hours and analyzed using Western Blotting with the indicated antibodies. Cell viability was measured using the MTT assay at 72 hours. Data are presented as mean ± SD of triplicate experiments. Statistical significance was determined using Student’s *t* test (**p < 0.01, ***p < 0.001, ****p < 0.0001). BER, base excision repair; FA, Fanconi anemia; HR, homologous recombination; KO, knockout; MMR, mismatch repair; NHEJ, non–homologous end joining.

*ARID1A* encodes a key component of the BAF chromatin-remodeling complex, which regulates chromatin structure and controls gene expression.^14^ This complex is involved in several critical pathways, including the AKT signaling pathway, reactive oxygen species production, apoptosis, and ATR-mediated DNA damage repair.^15^ To investigate the pathways disrupted in *ARID1A*-mutant osimertinib-resistant cells, we performed comprehensive gene expression and pathway analyses comparing PC9 parental cells with ARID1A-KO#7 cells. Among the pathways analyzed, cell cycle-associated genes exhibited the most significant alterations (Fig. 3A). ARID1A-KO#7 cells exhibited a substantial reduction in the expression of these genes compared with parental PC9 cells (Fig. 3B). In particular, genes involved in DNA repair pathways, including homologous recombination (HR) and the Fanconi anemia pathway, reported decreased expression in ARID1A-KO#7 and osimertinib-resistant PC9-OR#3 cells relative to parental PC9 cells (Fig. 3C). Therefore, we hypothesize that targeting DNA repair and cell cycle pathways can be an effective strategy to overcome resistance. To identify potential therapeutic targets for *ARID1A* mutation-associated osimertinib resistance, we performed a CRISPR knockout (KO) screening using a CRISPR RNA (crRNA) library targeting 746 protein kinase genes (Fig. 3D). In PC9-ARID1A-KO#7 cells, KO of 10 specific genes reduced cell viability by greater than 70%, with WEE1 KO exerting the most pronounced effect (Fig. 3E). Among the top 10 genes identified, seven were associated with cell cycle regulation, namely, *WEE1*, *PLK1*, *CDK11B*, *CHK1*, *BUB1B*, *PRPF4B*, and *AURKA* (Fig. 3E and F). WEE1, a critical regulator of the G2/M cell cycle checkpoint, is the most promising target.^16,17^ To validate these findings, we knocked down WEE1 expression in PC9, PC9-ARID1A-KO#7, and PC9-OR#3 cells using *WEE1*-specific siRNAs. WEE1 knockdown significantly suppressed the growth of both PC9-ARID1A-KO#7 and PC9-OR#3 cells, with a stronger inhibitory effect than in parental PC9 cells (Fig. 3G). Importantly, this growth suppression was not observed in at least two normal fibroblast cells (IMR-90 and MRC-5), suggesting a degree of specificity (Supplementary Fig. 4). These results indicate that *ARID1A* mutations induce significant changes in the expression of cell cycle-associated genes and suggest WEE1 as a potential therapeutic target for *ARID1A*-mutated, osimertinib-resistant *EGFR*-mutant NSCLC cells.

### WEE1 Inhibitors Enhanced Apoptosis in ARID1A-Mutant Osimertinib-Resistant Cells

**Figure 4.**
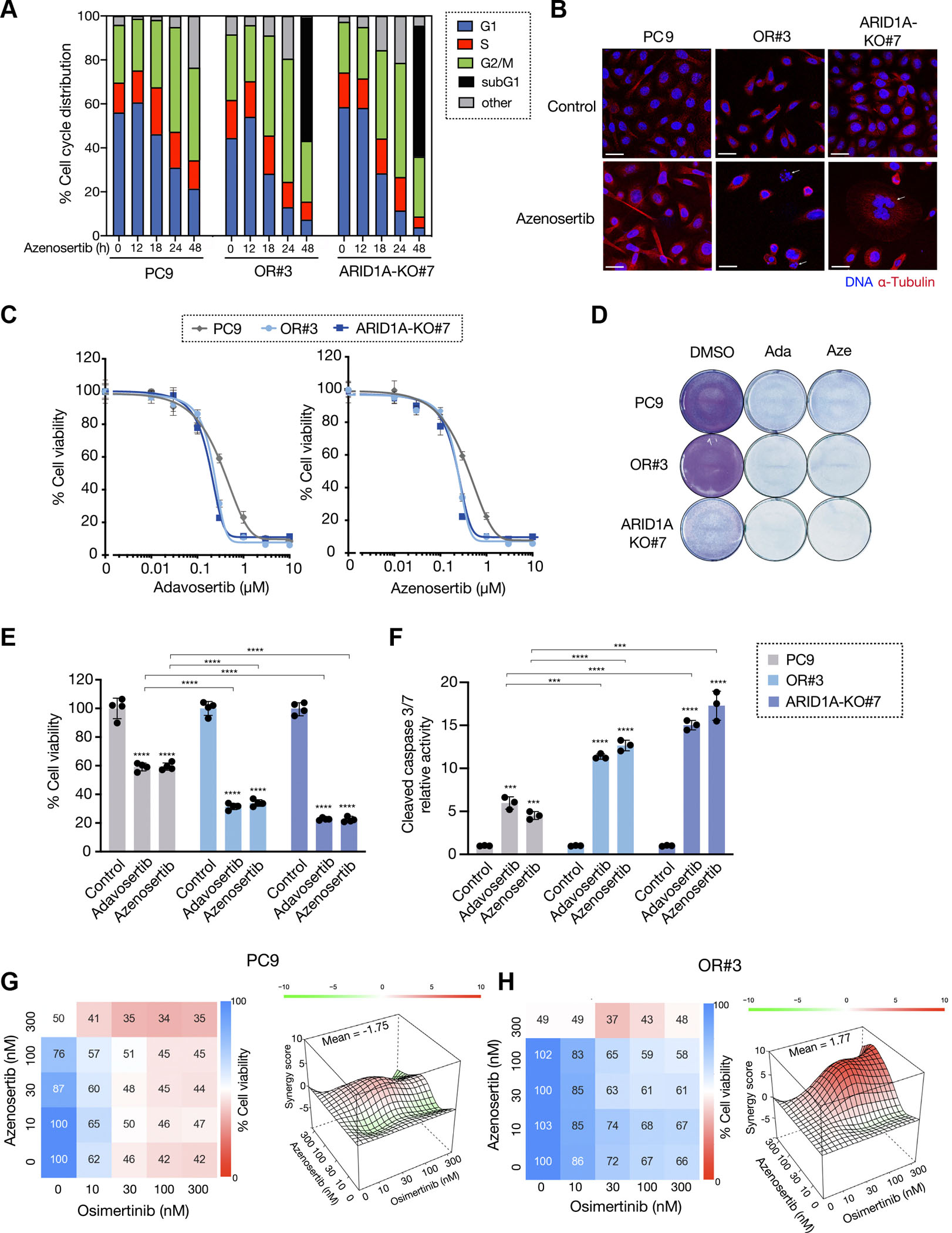
WEE1 inhibitors enhance apoptosis in *ARID1A*-mutant osimertinib-resistant cells. (*A*) PC9, OR#3, and ARID1A-KO#7 cells were treated with azenosertib (1 *μ*M), and cell cycle analysis was performed at the indicated time points using Deep Blue staining. (*B*) PC9, OR#3, and ARID1A-KO#7 cells were treated with 1 *μ*M azenosertib for 48 hours, fixed, and stained for a-tubulin (red) using immunofluorescence and for DNA using 4’,6-diamidino-2-phenylindole (DAPI) (blue). Mitotic catastrophe was evaluated by analyzing nuclear morphology under a confocal microscope. Representative images of multinucleated cells (indicated by white arrows) are presented. Scale bar: 30 *μ*m. (*C*) PC9, OR#3, and ARID1A-KO#7 cells were treated with various concentrations of adavosertib or azenosertib, and cell viability was assessed using an MTTassay. Bars represent the mean ± SD of triplicate experiments. (*D*) Cell growth after 7 days of treatment with 1 *μ*M osimertinib was analyzed using crystal violet staining. (*E*) PC9, OR#3, and ARID1A-KO#7 cells were treated with 1 *μ*M osimertinib for 72 hours, and cell viability was assessed using an MTT assay. Data are presented as mean ± SD of triplicate experiments. Statistical significance was determined using the Student’s *t* test (****p < 0.0001). (*F*) Apoptosis levels were quantified using the Caspase-Glo 3/7 assay. Bars represent the mean ± SD of triplicate experiments. Statistical significance was determined using the Student’s *t* test (***p < 0.001, ****p < 0.0001). (*G, H*) PC9 and OR#3 cells were treated with azenosertib and osimertinib for 72 hours at the indicated concentrations. Cell viability was assessed using an MTT assay. The two-dimensional surface response for cell inhibition and the three-dimensional surface Bliss synergy response are indicated. Data represent the mean of triplicates.

The WEE1 inhibitor adavosertib is currently under clinical evaluation,^18^ reporting promising efficacy but limited tolerability, likely due to poor kinase selectivity. To address this, a more selective WEE1 inhibitor, azenosertib (ZN-c3), has been developed,^19^ indicating higher kinase selectivity than adavosertib. Moreover, a phase I dose-escalation trial in patients with advanced solid tumors reported early signals of clinical activity for azenosertib.^20^ Given that WEE1 regulates the G2/M cell cycle checkpoint,^16^ we conducted a cell cycle assay using Deep Red staining and flow cytometry and revealed that azenosertib induced G2/M arrest and increased sub-G1 phase populations in PC9-OR#3 and PC9-ARID1A-KO#7 cells, indicative of apoptosis. This effect was not observed in parental PC9 cells (Fig. 4A). Furthermore, confocal microscopy revealed the formation of multinucleated cells in PC9-OR#3 and PC9-ARID1A-KO#7 after azenosertib treatment (Fig. 4B), suggesting that WEE1 inhibition induced mitotic catastrophe in these resistant cells.

To further assess the efficacy of WEE1 inhibitors, we determined the IC_50_ after 72 hours of treatment. Adavosertib and azenosertib reduced cell viability in a dose-dependent manner, with lower IC_50_ values observed in PC9-OR#3 and PC9-ARID1A-KO#7 cells than in parental PC9 cells. The inhibitory effects of adavosertib and azenosertib were comparable (Fig. 4C and E). A cell growth assay performed after an additional week of treatment produced consistent results (Fig. 4D). Notably, resistant cell lines exhibited significant activation of the apoptosis marker cleaved caspase-3/7, whereas parental PC9 cells exhibited only a modest increase (Fig. 4F).

We also evaluated the combined effects of WEE1 inhibitors and osimertinib using a multidimensional two-drug synergy assay analyzed with the Bliss independence model and found a synergistic effect in PC9-OR#3 cells but not in parental PC9 cells (Fig. 4G and H). Furthermore, combined treatment significantly enhanced apoptosis in PC9-OR#3 cells (Supplementary Fig. 5). These findings highlight the therapeutic potential of WEE1 inhibitors in enhancing apoptosis in *ARID1A*-mutant osimertinib-resistant cells.

### WEE1 Inhibitors Induced DNA Damage in ARID1A-Mutant Cells

**Figure 5.**
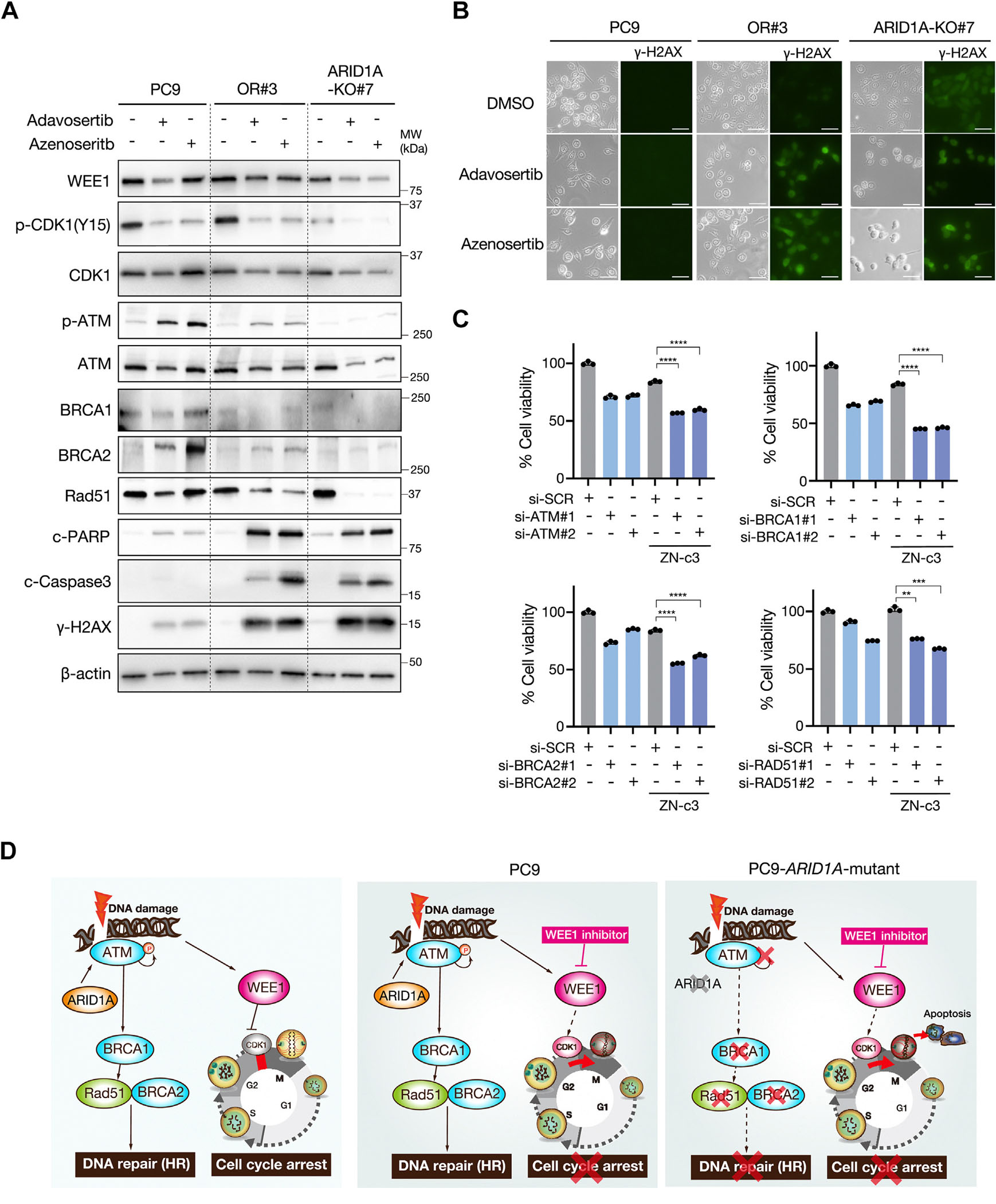
Induction of DNA damage by WEE1 inhibitors in *ARID1A*-mutant cells. (*A*) PC9, OR#3, and ARID1A-KO#7 cells were treated with 1 *μ*M adavosertib or azenosertib for 48 hours. Cell lysates were analyzed using Western Blotting with the indicated antibodies. (*B*) Immunofluorescence staining of H358 cells treated with 1 *μ*M adavosertib or 1 *μ*M azenosertib, using γH2AX-Alexa 488 (green) and DAPI (blue). Scale bars: 50 *μ*m. (*C*) Cell viability of PC9 cells transfected with small interfering RNAs targeting *ATM*, *BRCA1*, *BRCA2*, or *RAD51* for 72 hours was assessed using an MTT assay. Data are presented as mean ± SD of triplicate experiments. Statistical significance was determined using Student’s *t* test (**p < 0.01, ***p < 0.001, ****p < 0.0001). (*D*) Schematic representation of the proposed roles of ARID1A and WEE1 in *ARID1A*-mutant cells. si-SCR, scrambled control small interfering RNA.

To elucidate the underlying mechanisms, we evaluated the DNA damage response (DDR) pathway in cells treated with WEE1 inhibitors. In parental PC9 cells, WEE1 inhibition increased ATM phosphorylation, a key sensor of DNA damage. In contrast, PC9-OR#3 cells revealed only a slight increase, whereas ARID1A-KO#7 cells exhibited no significant changes (Fig. 5A). Similarly, the expression of the DNA repair component BRCA2 was increased in parental PC9 cells after WEE1 inhibition, whereas PC9-OR#3 cells indicated only a slight increase, and ARID1A-KO#7 cells exhibited no significant changes (Fig. 5A). Consistently, the expression levels of BRCA1 and RAD51 were reduced in these resistant cells whereas they remained stable in parental PC9 cells (Fig. 5A). Markers of DNA damage (γ-H2AX) and apoptosis (cleaved-PARP and cleaved-Caspase3) were significantly elevated in resistant cells after WEE1 inhibition (Fig. 5A). Immunocytochemistry further confirmed that WEE1 inhibitors increased γ-H2AX activity in resistant cells. Notably, baseline γ-H2AX levels were higher in ARID1A-KO#7 cells than in parental PC9 cells, indicating accumulated DNA damage probably caused by *ARID1A* deficiency (Fig. 5B).

To determine whether DNA repair components contribute to sensitivity to WEE1 inhibition, we knocked down *ATM*, *BRCA1*, *BRCA2*, and *RAD51* in parental PC9 cells and treated them with the WEE1 inhibitor azenosertib. Knockdown of *ATM*, *BRCA1*, or *BRCA2* significantly enhanced the effects of WEE1 inhibition (Fig. 5C). Although WEE1 inhibition promoted G2/M cell cycle progression, DNA repair components, including ATM, BRCA1, BRCA2, and RAD51, were activated to facilitate DNA repair, enabling cancer cell survival. Nevertheless, in *ARID1A*-mutant cells, the expression of cell cycle-related genes, including those involved in DNA repair, was decreased. This rendered *ARID1A*-mutant cells more susceptible to WEE1 inhibition, leading to an accumulation of DNA damage and subsequent apoptosis (Fig. 5D).

### WEE1 Inhibition Overcame Osimertinib Resistance in the LMC Model

**Figure 6.**
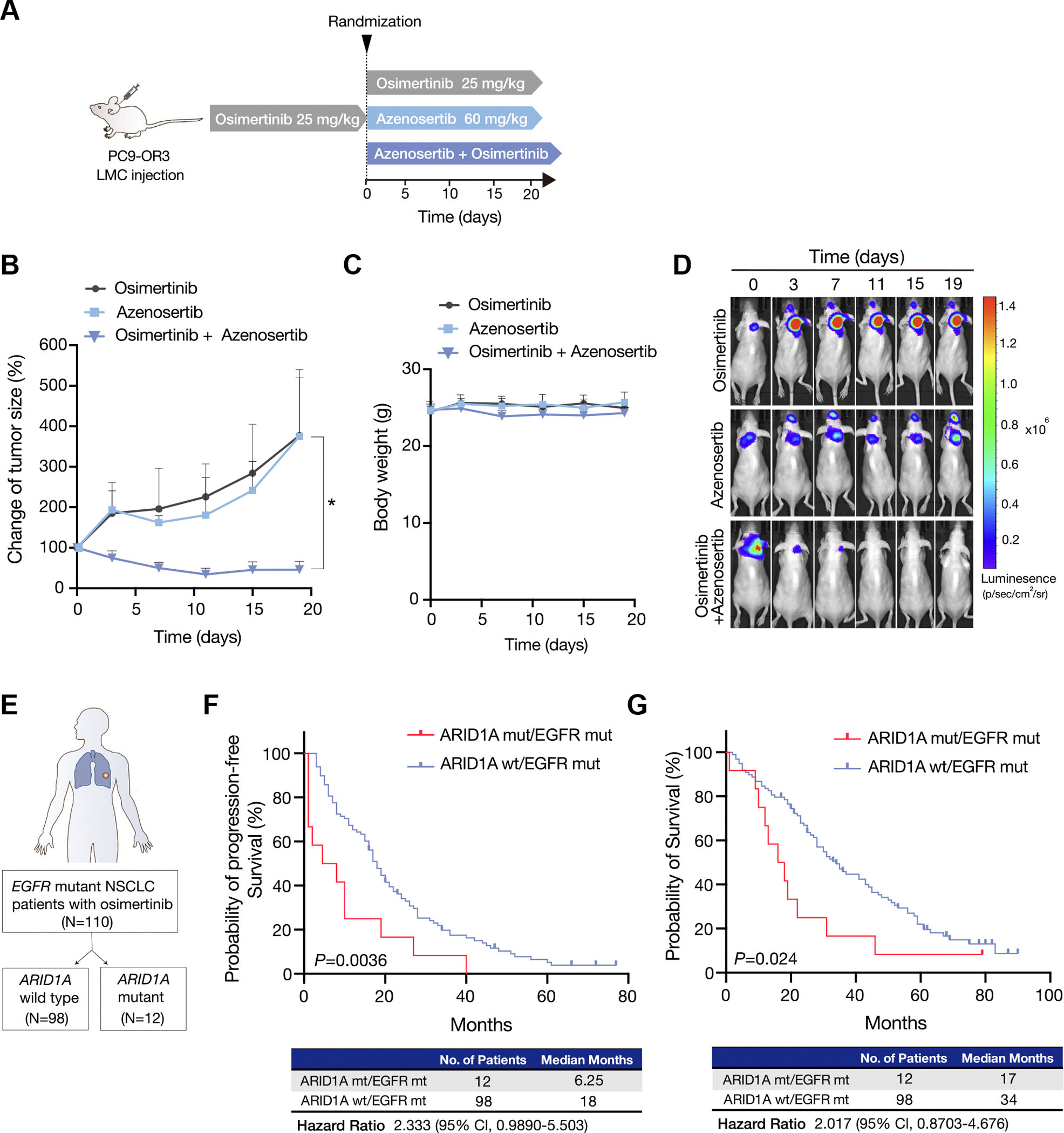
WEE1 inhibition enhances the therapeutic efficacy of osimertinib in LMC models and analysis of samples from patients with *EGFR*-mutant NSCLC. (*A*) Schematic representation of the in vivo experimental protocol used in the OR#3 LMC model. (*B*) Quantification of luminescence signals in mice treated daily with osimertinib (25 mg/kg, *n* = 8), azenosertib (60 mg/kg, *n* = 8), or a combination of azenosertib (60 mg) and osimertinib (25 mg/kg) until the conclusion of the experiment. Error bars represent the standard error of the mean. (*C*) Percentage change in body weight of mice throughout the treatment period. (*D*) Representative luminescence images of mice from each treatment group. (*E*) Schematic overview of the analysis performed on clinical samples from patients with *EGFR*-mutant NSCLC. (*F*) Kaplan–Meier analysis of progression-free survival in patients with *EGFR*-mutant lung cancer treated with osimertinib (*n* = 110) from the UCSF Tumor Bank, stratified by *ARID1A* mutation status. Statistical significance was assessed using the log-rank test. (*G*) Kaplan–Meier analysis of OS in patients with *EGFR*-mutant lung cancer treated with osimertinib (*n* = 110) from the UCSF Tumor Bank, stratified by *ARID1A* mutation status. Survival time was measured from osimertinib initiation. Statistical significance was assessed using the log-rank test. CI, confidence interval; mut, mutation.

To assess the in vivo therapeutic potential of WEE1 inhibition, we used a mouse model of LMC. PC9-OR#3 cells were injected into the leptomeningeal space of SHO mice and treated with osimertinib (25 mg/kg) for 14 days. Tumor progression confirmed osimertinib resistance in this model. After randomization, mice were treated with azenosertib (30 mg/kg), osimertinib (25 mg/kg), or a combination of both. Tumor size and body weight were monitored over the subsequent 20 days (Fig. 6A). Azenosertib monotherapy indicated limited efficacy, causing slight tumor suppression initially, but tumor progression resumed after 2 weeks of treatment (Fig. 6B and D). In contrast, the combination therapy significantly reduced tumor size, with no notable toxicity or weight loss observed (Fig. 6B–D). These results indicate that WEE1 inhibition, in combination with osimertinib, can effectively overcome *ARID1A* mutation-associated osimertinib resistance in the LMC model.

### Analysis in Patients With EGFR-Mutant NSCLC Treated With Osimertinib

To investigate the relationship between *ARID1A* mutations and osimertinib efficacy in patients with *EGFR*-mutant NSCLC, we analyzed clinical next-generation sequencing data from the University of California San Francisco (UCSF) osimertinib-treated patient cohort. *ARID1A* mutations were identified in 12 individuals, whereas 98 patients harbored wild-type *ARID1A* (Fig. 6E), most of which had *EGFR* exon-19 deletions (eight cases), whereas three patients carried *EGFR*-L858R mutations, revealing the predominance of *EGFR* exon-19 deletions in this cohort. In contrast, *ARID1A* mutations exhibited notable heterogeneity (Supplementary Table 1). Patients with *ARID1A* mutations indicated a significantly shorter median PFS of 6.25 months than 18.0 months for those with wild-type *ARID1A* (*p* = 0.0036) (Fig. 6F). This poor response was also reflected in OS, with 17.0 months in patients with *ARID1A* mutations versus 34.0 in the wild-type group (*p* = 0.024) (Fig. 6G). Moreover, CNS metastases were observed in seven of the 12 *ARID1A*-mutant cases. These findings highlight the critical role of *ARID1A* mutations in predicting resistance to osimertinib treatment and reduced efficacy in patients with *EGFR*-mutant NSCLC.

## Discussion

The advent of EGFR inhibitors, including osimertinib, has markedly improved the treatment of *EGFR*-mutant NSCLC; nevertheless, achieving curative outcomes remains challenging owing to treatment resistance, particularly in the context of CNS metastases. In this study, we identified *ARID1A* mutations as a critical driver of osimertinib resistance in *EGFR*-mutant NSCLC, specifically within an LMC model. Our CRISPR-Cas9 gene KO screenings found that WEE1 inhibition effectively induced apoptosis in *ARID1A*-mutant, osimertinib-resistant cells, highlighting a promising therapeutic strategy that leverages ARID1A-associated vulnerabilities.

The SWI/SNF chromatin remodeling complex, in which ARID1A is a frequently mutated subunit, is pivotal in maintaining genomic stability and regulating diverse cellular functions.^21^ In lung cancer, *ARID1A* mutations have been linked to poor patient survival and increased metastatic potential.^22^ Loss of ARID1A expression is linked to enhanced metastasis and poor prognosis, partly owing to its regulatory role in pathways, such as Akt/mTOR.^23^ Specifically, ARID1A suppresses the Akt/mTOR pathway by inhibiting phosphoinositide-3-kinase interacting protein 1.^24,25^ In breast cancer, *ARID1A* mutations have been implicated in trastuzumab resistance through Akt pathway activation.^26^ Interestingly, our study revealed a different mechanism in *ARID1A*-mutant osimertinib-resistant cells. Indeed, these cells exhibited significant activation of the MAPK pathway, with no substantial changes in Akt signaling. Recent evidence suggests that ARID1A loss can activate MAPK signaling through the downregulation of DUSP4, potentially owing to impaired chromatin remodeling.^27^ This finding aligns with our observation that MEK inhibitors reduced the viability of *ARID1A*-mutant cells, underscoring the potential of MAPK pathway inhibition as a therapeutic approach. Nevertheless, the precise mechanisms by which *ARID1A* mutations regulate MAPK signaling and contribute to osimertinib resistance remain to be elucidated.

WEE1, a kinase crucial for regulating the G2/M checkpoint, is vital in cell cycle control and DNA repair.^16^ On DNA damage, WEE1 is activated by the ATR-CHK1 cascade, which phosphorylates CDK1 at Tyr15, preventing cells from entering mitosis during the G2/M phase.^16,17,28^ Our *ARID1A*-deficient cells revealed reduced expression of DDR-associated genes, indicating a compromised DDR and heightened sensitivity to WEE1 inhibition. This finding aligns with those of previous studies reporting that *ARID1A*-deficient triple-negative breast cancer cells are highly sensitive to ATR inhibition owing to impaired DDR function.^29^ Moreover, combining DDR inhibitors, such as ATR or WEE1 inhibitors, with BRD4 inhibitors exerts synergistic effects in reducing survival in *ARID1A*-mutant ovarian carcinoma cells by further impairing homologous recombination-mediated double-strand break repair.^30^ In addition, ARID1A has been implicated in repairing DNA double-strand breaks induced by etoposide through p-ATM activation in gastric cancer cells.^31^ Consistently, we found that p-ATM levels were not elevated in *ARID1A*-mutant cells, confirming DDR deficiencies in these cells. These results underscore the potential of exploiting synthetic lethality by targeting WEE1 in *ARID1A*-mutant, osimertinib-resistant NSCLC.

In addition to targeting WEE1, azenosertib also strongly inhibits PLK1/2/3, with near identical potency to its effect on WEE1, at least for PLK2.^19^ Our CRISPR KO screening identified PLK1 as the second top-priority gene in the PC9-ARID1A-KO#7 cells (Fig. 3D), suggesting that a portion of the antitumor activity observed in this study may be attributable to WEE1 and PLK1/2/3 inhibitions. PLK1/2/3 play critical roles in mitotic regulation; therefore, their inhibition may be an on-target effect rather than an unintended off-target phenomenon. Further investigations remain warranted to clarify whether the anticancer efficacy of azenosertib is attributed to the inhibition of WEE1, PLK1/2/3, or a combination thereof.

WEE1 inhibitors have indicated encouraging preclinical and clinical results across various solid tumors, including ovarian, endometrial, breast, and pancreatic cancers.^18,32^ For instance, adavosertib has undergone assessments in clinical settings, including a phase II study combined with carboplatin in *TP53*-mutant ovarian cancer and a phase I study with docetaxel in pancreatic cancer.^18,33^ Nevertheless, its clinical application has been limited by hematologic toxicities. To address this, a selective WEE1 inhibitor, azenosertib, was developed, revealing higher kinase selectivity than that of adavosertib.^19,34^ Our study highlights the potential of azenosertib, particularly in CNS-resistant cases of *EGFR*-mutant NSCLC. Although WEE1 inhibition alone exhibited limited efficacy, combining azenosertib with osimertinib significantly suppressed tumor growth in vivo. This is consistent with our earlier findings when azenosertib combined with a KRAS-G12C inhibitor led to tumor reduction in patient-derived xenograft models from patients with KRAS-G12C inhibitor resistance,^35^ suggesting that combination therapies targeting the DDR pathway are a promising approach to overcome resistance. A similar mechanism may apply in *EGFR*-mutant NSCLC, when combining azenosertib with osimertinib effectively targets osimertinib-resistant cells. Further studies and clinical trials evaluating the combination of WEE1 and EGFR inhibitors in patients with *ARID1A*-mutant NSCLC are warranted to confirm and expand on these findings. Clinical data on the safety of azenosertib relative to adavosertib remain limited, necessitating additional evaluation concerning its toxicity profile and potential advantages, especially given the recent concerns over serious adverse events in early-phase trials. Azenosertib’s higher kinase selectivity suggests a favorable safety profile; nevertheless, comprehensive comparative data, particularly regarding hematologic toxicity, are lacking, and the Food and Drug Administration’s partial clinical hold further underscores that the overall safety profile remains under active evaluation. Furthermore, although this study found that azenosertib exhibited clear synergy, other WEE1 inhibitors may also confer comparable benefits, which warrants further studies to clarify the differences in efficacy.

CNS metastases, including brain metastases and LMC, occur in 20% to 40% of patients with cancer and may lead to debilitating neurologic symptoms, thus significantly decreasing quality of life and correlating with poor prognosis. In this study, *ARID1A* mutations drive osimertinib resistance in *EGFR*-mutant NSCLC, particularly in an LMC model. Interestingly, *ARID1A* mutations are also common in glioblastoma, where they enhance malignancy and confer resistance to temozolomide.^36^ This underscores the role of ARID1A in CNS involvement; nevertheless, *ARID1A* mutations are not exclusive to CNS disease. Their impairment of DDR and cell cycle regulation could similarly promote resistance in pulmonary or other systemic recurrences of *EGFR*-mutant NSCLC. Indeed, our clinical data revealed *ARID1A* mutations in primary lung tumors and metastatic samples, suggesting a broader impact on osimertinib efficacy beyond the CNS. To clarify whether *ARID1A* mutations are predominantly associated with the CNS or occur at comparable frequencies in other metastatic sites, we analyzed clinical next-generation sequencing data from 110 patients with *EGFR*-mutant NSCLC at the UCSF Tumor Bank and identified *ARID1A* mutations in 12 samples (11%) associated with significantly shorter PFS. Notably, CNS metastases were observed in seven of these 12 patients, suggesting that *ARID1A* mutations arise or gain relevance after CNS metastasis. Our OS analysis further indicated that the worse outcome in patients with *ARID1A* mutations became more evident after osimertinib therapy initiation (Fig. 6G), implying that *ARID1A* mutations exert a greater impact once EGFR-directed treatment is under way. CNS metastases may also exacerbate resistance mechanisms, reducing PFS in patients with *ARID1A* mutations.

Interestingly, *ARID1A* mutations were identified in five patients with primary lung tumors, and eight of 12 patients with *ARID1A* mutations carried these mutations before initiating osimertinib, indicating a potential role in intrinsic resistance. Although our osimertinib-acquired resistance model reported that *ARID1A* deletion conferred resistance, these findings suggest that *ARID1A* mutations contribute to intrinsic and acquired resistance. This dual role is consistent with findings in colorectal cancer, in which *ARID1A* mutations confer both intrinsic and acquired resistance to cetuximab.^37^ These results underscore the complex role of *ARID1A* mutations in osimertinib resistance, highlighting their potential as prognostic biomarkers for predicting osimertinib efficacy. Although *ARID1A* mutations often induce loss of function,^38^ variability in patient outcomes suggests that specific variants drive resistance through distinct mechanisms. Future studies should identify these variants and tailor therapeutic approaches accordingly.

*EGFR*-mutant NSCLC typically exhibits limited responsiveness to immune checkpoint inhibitors (ICIs), partly owing to a low tumor mutation burden and an immunosuppressive tumor microenvironment.^39^ *ARID1A* mutations enhance antitumor immunity in specific contexts by modulating neoantigen presentation or tumor-infiltrating lymphocytes.^40^ Indeed, an analysis in patients with *ARID1A*-mutant NSCLC found that *ARID1A* mutations were more sensitive to ICI therapy,^41^ suggesting that *ARID1A*-deficient tumors benefit from ICI-based treatments, in addition to WEE1 inhibitors. Further investigation on the interplay among *ARID1A* mutations, EGFR signaling, and tumor immunogenicity remains warranted.

This study has several strengths. It integrated in vitro and in vivo models, including an LMC model, to investigate the role of *ARID1A* mutations in osimertinib resistance. The use of CRISPR-Cas9 screening identified WEE1 as a therapeutic target, providing mechanistic insights and highlighting a potential strategy for overcoming resistance. This study also has some limitations. First, biopsies were collected before and after osimertinib treatment (Supplementary Table 1), potentially confounding the assessment of *ARID1A* mutation status in primary versus acquired resistance. Second, our PFS and OS analyses were based on the date of osimertinib initiation. Some patients began osimertinib as first-line therapy, whereas others received it in later lines. Although this variation in treatment lines might have influenced the analysis, the proportion of first-line usage was similar in the groups (Fisher’s exact test; *p* = 1.00) (Supplementary Table 2). Third, our findings were based on preclinical models, which may not fully recapitulate human tumor complexity. Finally, the molecular mechanisms linking *ARID1A* mutations to MAPK activation and DDR deficiencies require further exploration.

In conclusion, our findings reveal the involvement of *ARID1A* mutations in osimertinib resistance in *EGFR*-mutant NSCLC, with particular relevance to CNS metastases. The multifaceted nature of *ARID1A* mutations, contributing to resistance while simultaneously creating vulnerabilities in DNA damage repair pathways, renders *ARID1A*-mutant cells particularly susceptible to WEE1 inhibition. Clinical validation of selective WEE1 inhibitors, especially in combination with EGFR inhibitors, is crucial for optimizing personalized treatment strategies and improving outcomes for patients with *EGFR*-mutant NSCLC.

## Supplementary Material

1

Supplementary Data

Note: To access the supplementary material accompanying this article, visit the online version of the *Journal of Thoracic Oncology* at www.jto.org and at https://doi.org/10.1016/j.jtho.2025.06.007.

## Data Availability

The data sets generated and analyzed during this study are available from the corresponding author upon request for reanalyzing the data presented in this report. Additional information required to facilitate data reanalysis is accessible from the Lead Contact on request.

## References

[1] Barnholtz-Sloan JS, Sloan AE, Davis FG, Vigneau FD, Lai P, Sawaya RE. Incidence proportions of brain metastases in patients diagnosed (1973 to 2001) in the Metropolitan Detroit Cancer Surveillance System. J Clin Oncol. 2004;22:2865–2872. 15254054 10.1200/JCO.2004.12.149

[2] Iuchi T, Shingyoji M, Itakura M, Frequency of brain metastases in non-small-cell lung cancer, and their association with epidermal growth factor receptor mutations. Int J Clin Oncol. 2015;20:674–679. 25336382 10.1007/s10147-014-0760-9

[3] Li YS, Jiang BY, Yang JJ, Leptomeningeal metastases in patients with NSCLC with EGFR mutations. J Thorac Oncol. 2016;11:1962–1969. 27539328 10.1016/j.jtho.2016.06.029

[4] Rosell R, Carcereny E, Gervais R, Erlotinib versus standard chemotherapy as first-line treatment for European patients with advanced EGFR mutation-positive non-small-cell lung cancer (EURTAC): a multicentre, open-label, randomised phase 3 trial. Lancet Oncol. 2012;13:239–246. 22285168 10.1016/S1470-2045(11)70393-X

[5] Minari R, Bordi P, Tiseo M. Third-generation epidermal growth factor receptor-tyrosine kinase inhibitors in T790M-positive non-small cell lung cancer: review on emerged mechanisms of resistance. Transl Lung Cancer Res. 2016;5:695–708. 28149764 10.21037/tlcr.2016.12.02 PMC5233880

[6] Soria JC, Ohe Y, Vansteenkiste J, Osimertinib in untreated *EGFR*-mutated advanced non–small-cell lung cancer. N Engl J Med. 2018;378:113–125. 29151359 10.1056/NEJMoa1713137

[7] Ramalingam SS, Vansteenkiste J, Planchard D, Overall survival with osimertinib in untreated, EGFR-mutated advanced NSCLC. N Engl J Med. 2020;382:41–50. 31751012 10.1056/NEJMoa1913662

[8] Erickson AW, Brastianos PK, Das S. Assessment of effectiveness and safety of osimertinib for patients with intracranial metastatic disease: a systematic review and meta-analysis. JAMA Netw Open. 2020;3:e201617. 32211870 10.1001/jamanetworkopen.2020.1617 PMC7097701

[9] Arulananda S, Do H, Rivalland G, Standard dose osimertinib for erlotinib refractory T790M-negative *EGFR*-mutant non-small cell lung cancer with leptomeningeal disease. J Thorac Dis. 2019;11:1756–1764. 31285867 10.21037/jtd.2019.05.41 PMC6588760

[10] Fukuda K, Takeuchi S, Arai S, Glycogen synthase kinase-3 inhibition overcomes epithelial-mesenchymal transition-associated resistance to osimertinib in EGFR-mutant lung cancer. Cancer Sci. 2020;111:2374–2384. 32391602 10.1111/cas.14454 PMC7385349

[11] Fukuda K, Takeuchi S, Arai S, Epithelial-to-mesenchymal transition is a mechanism of ALK inhibitor resistance in lung cancer independent of *ALK* mutation status. Cancer Res. 2019;79:1658–1670. 30737231 10.1158/0008-5472.CAN-18-2052

[12] Fu K, Xie F, Wang F, Fu L. Therapeutic strategies for *EGFR*-mutated non-small cell lung cancer patients with osimertinib resistance. J Hematol Oncol. 2022;15:173. 36482474 10.1186/s13045-022-01391-4 PMC9733018

[13] Fukuda K, Otani S, Takeuchi S, Trametinib overcomes *KRAS*-G12V-induced osimertinib resistance in a leptomeningeal carcinomatosis model of *EGFR*-mutant lung cancer. Cancer Sci. 2021;112:3784–3795. 34145930 10.1111/cas.15035 PMC8409422

[14] Mathur R ARID1A loss in cancer: towards a mechanistic understanding. Pharmacol Ther. 2018;190:15–23. 29730444 10.1016/j.pharmthera.2018.05.001

[15] Caumanns JJ, Wisman GBA, Berns K, van der Zee AGJ, de Jong S. *ARID1A* mutant ovarian clear cell carcinoma: a clear target for synthetic lethal strategies. Biochim Biophys Acta Rev Cancer. 2018;1870:176–184. 30025943 10.1016/j.bbcan.2018.07.005

[16] Russell P, Nurse P. Negative regulation of mitosis by wee1+, a gene encoding a protein kinase homolog. Cell. 1987;49:559–567. 3032459 10.1016/0092-8674(87)90458-2

[17] Parker LL, Piwnica-Worms H. Inactivation of the p34cdc2-cyclin B complex by the human WEE1 tyrosine kinase. Science. 1992;257:1955–1957. 1384126 10.1126/science.1384126

[18] Kong A, Mehanna H. WEE1 inhibitor: clinical development. Curr Oncol Rep. 2021;23:107. 34269904 10.1007/s11912-021-01098-8 PMC8285350

[19] Huang PQ, Boren BC, Hegde SG, Discovery of ZN-c3, a highly potent and selective Wee1 inhibitor undergoing evaluation in clinical trials for the treatment of cancer. J Med Chem. 2021;64:13004–13024. 34423975 10.1021/acs.jmedchem.1c01121

[20] Tolcher A, Mamdani H, Chalasani P, Abstract CT016: Clinical activity of single-agent ZN-c3, an oral WEE1 inhibitor, in a phase 1 dose-escalation trial in patients with advanced solid tumors. Cancer Res. 2021;81:CT016.

[21] Mullen J, Kato S, Sicklick JK, Kurzrock R. Targeting ARID1A mutations in cancer. Cancer Treat Rev. 2021;100:102287. 34619527 10.1016/j.ctrv.2021.102287

[22] Sun D, Tian L, Zhu Y, Subunits of ARID1 serve as novel biomarkers for the sensitivity to immune checkpoint inhibitors and prognosis of advanced non-small cell lung cancer. Mol Med. 2020;26:78. 32791957 10.1186/s10020-020-00208-9 PMC7425138

[23] Sun D, Zhu Y, Zhao H, Loss of ARID1A expression promotes lung adenocarcinoma metastasis and predicts a poor prognosis. Cell Oncol (Dordr). 2021;44:1019–1034. 34109546 10.1007/s13402-021-00616-x PMC12980766

[24] Rehman H, Chandrashekar DS, Balabhadrapatruni C, ARID1A-deficient bladder cancer is dependent on PI3K signaling and sensitive to EZH2 and PI3K inhibitors. JCI Insight. 2022;7:e155899. 35852858 10.1172/jci.insight.155899 PMC9462490

[25] Jin F, Yang Z, Shao J, ARID1A mutations in lung cancer: biology, prognostic role, and therapeutic implications. Trends Mol Med. 2023;29:646–658. 37179132 10.1016/j.molmed.2023.04.005

[26] Berns K, Sonnenblick A, Gennissen A, Loss of ARID1A activates ANXA1, which serves as a predictive biomarker for trastuzumab resistance. Clin Cancer Res. 2016;22:5238–5248. 27172896 10.1158/1078-0432.CCR-15-2996

[27] Mandal J, Yu ZC, Shih IM, Wang TL. ARID1A loss activates MAPK signaling via DUSP4 downregulation. J Biomed Sci. 2023;30:94. 38071325 10.1186/s12929-023-00985-5 PMC10709884

[28] O’Connor MJ. Targeting the DNA damage response in cancer. Mol Cell. 2015;60:547–560. 26590714 10.1016/j.molcel.2015.10.040

[29] Williamson CT, Miller R, Pemberton HN, ATR inhibitors as a synthetic lethal therapy for tumours deficient in ARID1A. Nat Commun. 2016;7:13837. 27958275 10.1038/ncomms13837 PMC5159945

[30] Simpkins F, Kinose Y, Xu H, Dual blockade of BRD4 and ATR/WEE1 pathways exploits ARID1A loss in clear cell ovarian cancer. https://www.researchsquare.com/article/rs-3314138/v1. Accessed June 30, 2025.

[31] Zhang Y, Qian HS, Hu G, Wang L, Zhu Y. ARID1A is involved in DNA double-strand break repair in gastric cancer. J Gastrointest Oncol. 2024;15:862–872. 38989399 10.21037/jgo-24-283 PMC11231857

[32] Takebe N, Naqash AR, O’Sullivan Coyne G, Safety, antitumor activity, and biomarker analysis in a phase I trial of the once-daily Wee1 inhibitor adavosertib (AZD1775) in patients with advanced solid tumors. Clin Cancer Res. 2021;27:3834–3844. 33863809 10.1158/1078-0432.CCR-21-0329 PMC8282703

[33] Leijen S, van Geel RM, Sonke GS, Phase II study of WEE1 inhibitor AZD1775 Plus carboplatin in patients with TP53-mutated ovarian cancer refractory or resistant to first-line therapy within 3 months. J Clin Oncol. 2016;34:4354–4361. 27998224 10.1200/JCO.2016.67.5942

[34] Jameson NM, Kim D, Lee C, The selective WEE1 inhibitor azenosertib shows synergistic antitumor activity with KRASG12C inhibitors in preclinical models. Cancer Res Commun. 2025;5:240–252. 39807828 10.1158/2767-9764.CRC-24-0411 PMC11795354

[35] Fukuda K, Takeuchi S, Arai S, Targeting WEE1 enhances the antitumor effect of KRAS-mutated non-small cell lung cancer harboring TP53 mutations. Cell Rep Med. 2024;5:101578. 38776912 10.1016/j.xcrm.2024.101578 PMC11228449

[36] Xiao M, Cui X, Xu C, Deep-targeted gene sequencing reveals ARID1A mutation as an important driver of glioblastoma. CNS Neurosci Ther. 2024;30:e14698. 38600891 10.1111/cns.14698 PMC11007544

[37] Johnson RM, Qu X, Lin CF, *ARID1A* mutations confer intrinsic and acquired resistance to cetuximab treatment in colorectal cancer. Nat Commun. 2022;13:5478. 36117191 10.1038/s41467-022-33172-5 PMC9482920

[38] Jiang T, Chen X, Su C, Ren S, Zhou C. Pan-cancer analysis of ARID1A alterations as biomarkers for immunotherapy outcomes. J Cancer. 2020;11:776–780. 31949479 10.7150/jca.41296 PMC6959029

[39] Al Bakir M, Reading JL, Gamble S, Clonal driver neoantigen loss under EGFR TKI and immune selection pressures. Nature. 2025;639:1052–1059. 39972134 10.1038/s41586-025-08586-y PMC11946900

[40] Shen J, Ju Z, Zhao W, ARID1A deficiency promotes mutability and potentiates therapeutic antitumor immunity unleashed by immune checkpoint blockade. Nat Med. 2018;24:556–562. 29736026 10.1038/s41591-018-0012-z PMC6076433

[41] Zhu G, Shi R, Li Y, *ARID1A, ARID1B*, and *ARID2* mutations serve as potential biomarkers for immune checkpoint blockade in patients with non-small cell lung cancer. Front Immunol. 2021;12:670040. 34512623 10.3389/fimmu.2021.670040 PMC8426508

