# Supplementary material for: Targeting WEE1 to Overcome ARID1A Mutation-Driven Osimertinib Resistance in EGFR-Mutant Lung Cancer
Source: J Thorac Oncol. Author manuscript; Available in PMC 2026 Jun 27. (PMC13310150; doi:10.1016/j.jtho.2025.06.007)
Supplement: 1 [file NIHMS2177626-supplement-1.pdf]

Figure S1

A

Cell	Flanking Sequence in ARID1A
PC9	No mutation
PC9-OR#3	CCATCTTCTTCCTTAGGCCG(delC)CAGGCTACCCCAATATGAAT
PC9-OR#3-Clone1	CCATCTTCTTCCTTAGGCCG(delC)CAGGCTACCCCAATATGAAT
PC9-OR#3-Clone2	CCATCTTCTTCCTTAGGCCG(delC)CAGGCTACCCCAATATGAAT
PC9-OR#3-Clone3	CCATCTTCTTCCTTAGGCCG(delC)CAGGCTACCCCAATATGAAT
PC9-OR#3-Clone4	CCATCTTCTTCCTTAGGCCG(delC)CAGGCTACCCCAATATGAAT

B

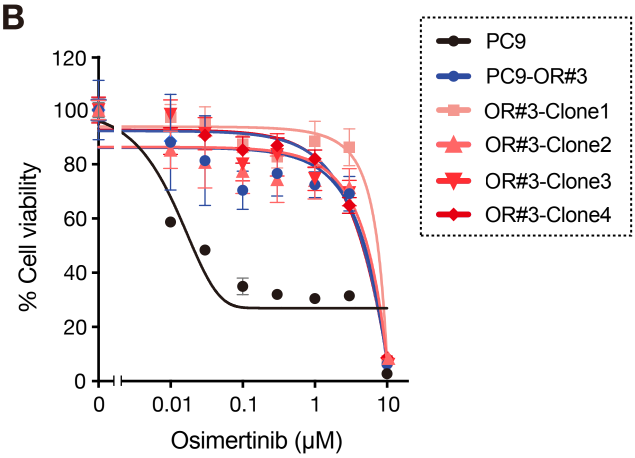

C

ARID1A

ARID domain | Pro913 | ARID1A antibody epitope

MAAQVAPAAASSLGNPPPPPPSELKKAEQQQREEAGGEAAAAAAAERGEMKAAAGQESEGPAVGPPQPLGKELQDGAESNGGGGGGGAGSGGGPGAEPDLKNSNGNAGPRPALNNNLTEPPGGGGGGSSDGV
GAPPHSAAAALPPPAYGFGQPYGRSPSAVAAAAAAVFHQQHGGQQSPGLAALQSGGGGGLEPYAGPQQNSHDHGFPNHQYNSYYPNRSAYPPPAPAYALSSPRGGTPGSGAAAAAGSKPPPSSSASASSSSSS
FAQQRFGAMGGGGPSAAGGGTPQPTATPTLNQLLTSPSSARGYQGYPGGDYSGGPQDGGAGKGPADMASQCWGAAAAAAAAAAASGGAQQRSHHAPMSPGSSGGGGQPLARTPQPSSPMDQMGKMRPQP
YGGTNPYSQQQGPPSGPQQGHGYPGQPYGSQTPQRYPMTMQGRAQSAMGGLSYTQQIPPYGQQGPSGYGQQGQTPYYNQQSPHPQQQQPPYSQQPPSQTPHAQPSYQQQPQSQPPQLQSSQPPYSQQPS
QPPHQQSPAPYPSQQSTTQQHPQSQPPYSQPQAQSPYQQQQPQQPAPSTLSQQAAYPQPQSQQSQQTAYSQQRFPPPQELSQDSFGSQASSAPSMTSSKGGQEDMNLSLQSRPSSLPDLSGSIDDLPMGTEG
ALSPGVSTSGISSSQGEQSNPAQSPFSPHTSPHLPGIRGPSPSPVGSPASVAQSRSGPLSPAAVPGNQMPPRPPSGQSDSIMHPSMNQSSIAQDRGYMQRNPQMPQYSSPQPGSALSPRQPSGGQIHTGMGSYQ
QNSMGSYGPQGGQYGPQGGYPRQPNYNALPNANYPSAGMAGGINPMGAGGQMHGQPGIPPYGTLPPGRMSHASMGNRPYGPNMANMPPQVGSGMCPPPGGMNRKTQETAVAMHVAANSIQNRPPGYPNM
NQGGMMGTGPPYGQGINSMAGMINPQGPPYSMGGTMANNSAGMAASPEMMGLGDVKLTPATKMNNKADGTPKTESKSKKSSSSTTTNEKITKLYELGGEPERKMWVDRYLAFTEEKAMGMTNLPAVGRKPLDL
YRLYVSVKEIGGLTQVNKNKKWRELATNLNVGTSSSAASSLKKQYIQCLYAFECKIERGEDPPPDIFAAADSKKSQPKIQPPSPAGSGSMQGPQTPQSTSSSMAEGGDLKPPTPASTPHSQIPPLPGMSRSNSVGIQD
AFNDGSDSTFQKRNSMTPNPGYQPSMNTSDMMGRMSYEPNKDPYGSMRKAPGSDPFMSSGQGPNGGMGDPYSRAAGPGLGNVAMGPRQHYPYGGPYDRVRTEPGIGPEGNMSTGAPQPNLMPSNPDSGM
YSPSRYPPQQQQQQQQRHDSYGNQFSTQGTPSGSPFPSQQTTMYQQQQQVSSPAPLPRPMENRTSPSKSPFLHSGMKMQKAGPPVPASHIAPAPVQPPMIRRDITFPPGSVEATQPVLKQRRRLTMKDIGTPEA
WRVMMSLKSGLLAESTWALDTINILLYDDNSIM*

ARID1A-Pro231del

Chaged Amino Acid

MAAQVAPAAASSLGNPPPPPPSELKKAEQQQREEAGGEAAAAAAAERGEMKAAAGQESEGPAVGPPQPLGKELQDGAESNGGGGGGGAGSGGGPGAEPDLKNSNGNAGPRPALNNNLTEPPGGGGGGSSDGV
GAPPHSAAAALPPPAYGFGQPYGRSPSAVAAAAAAVFHQQHGGQQSPGLAALQSGGGGGLEPYAGPQQNSHDHGFPNHQYNSYYPNRSAYPPPAPAYALSSPRGGTPGSGAAAAAGSKPPPSSSASASSSSSS
FAQQRFGAMGGGGPSAAGGGTPQPTATPTLNQLLTSPSSARGYQGYPGGDYSGGPQDGGAGKGPADMASQCWGAAAAAAAAAAASGGAQQRSHHAPMSPGSSGGGGQPLARTPQPSSPMDQMGKMRPQP
YGGTNPYSQQQGPPSGPQQGHGYPGQPYGSQTPQRYPMTMQGRAQSAMGGLSYTQQIPPYGQQGPSGYGQQGQTPYYNQQSPHPQQQQPPYSQQPPSQTPHAQPSYQQQPQSQPPQLQSSQPPYSQQPS
QPPHQQSPAPYPSQQSTTQQHPQSQPPYSQPQAQSPYQQQQPQQPAPSTLSQQAAYPQPQSQQSQQTAYSQQRFPPPQELSQDSFGSQASSAPSMTSSKGGQEDMNLSLQSRPSSLPDLSGSIDDLPMGTEG
ALSPGVSTSGISSSQGEQSNPAQSPFSPHTSPHLPGIRGPSPSPVGSPASVAQSRSGPLSPAAVPGNQMPPRPPSGQSDSIMHPSMNQSSIAQDRGYMQRNPQMPQYSSPQPGSALSPRQPSGGQIHTGMGSYQ
QNSMGSYGPQGGQYGPQGGYPRQPNYNALPNANYPSAGMAGGINPMGAGGQMHGQPGIPPYGTLPPGRMSHASMGNRPYGPNMANMPPQVGSGMCPPPGGMNRKTQETAVAMHVAANSIQNRP**QATPI***

D

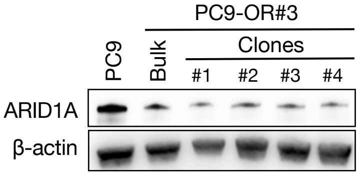

Figure S1 (A) Comparison of genomic DNA from PC9 parental cells, resistant lines, and the clone cells revealed the presence of ARID1A c2738del mutation.(B)PC9 parental cells and four clone cell lines were treated with indicated concentrations of osimertinib, and cell viability was measured using an MTT assay at 72 h. Bars represent mean ± SD of triplicate. (C)Location of ARID1A-Pro913(c2738) and the amino acid sequence of mutated protein (D) Cell lysates were analyzed by western blotting with the indicated antibodies.

Figure S2

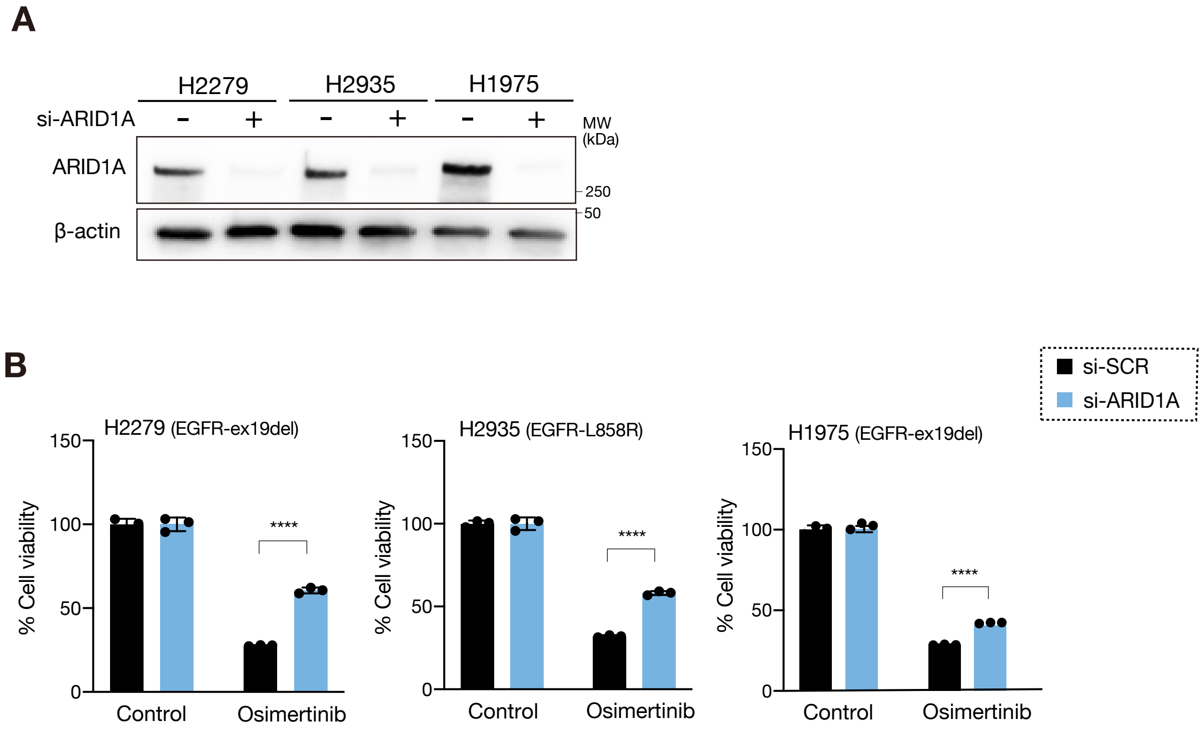

Figure S2 (A) H2279, H2935 and H1975 cells were transfected with siRNAs targeting ARID1A. Cell lysates were analyzed by western blotting with the indicated antibodies. (B) H2279, H2935 and H1975 cells were transfected with siRNAs targeting ARID1A and/or treated with 1 µM osimertinib. Cell viability was assessed using an MTT assay at 72 h. Bars represent mean ± SD of triplicate. Statistical significance was determined using Student's t test. ****$p < 0.0001$.

Figure S3

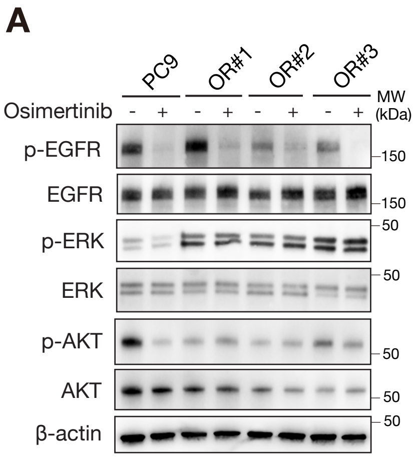

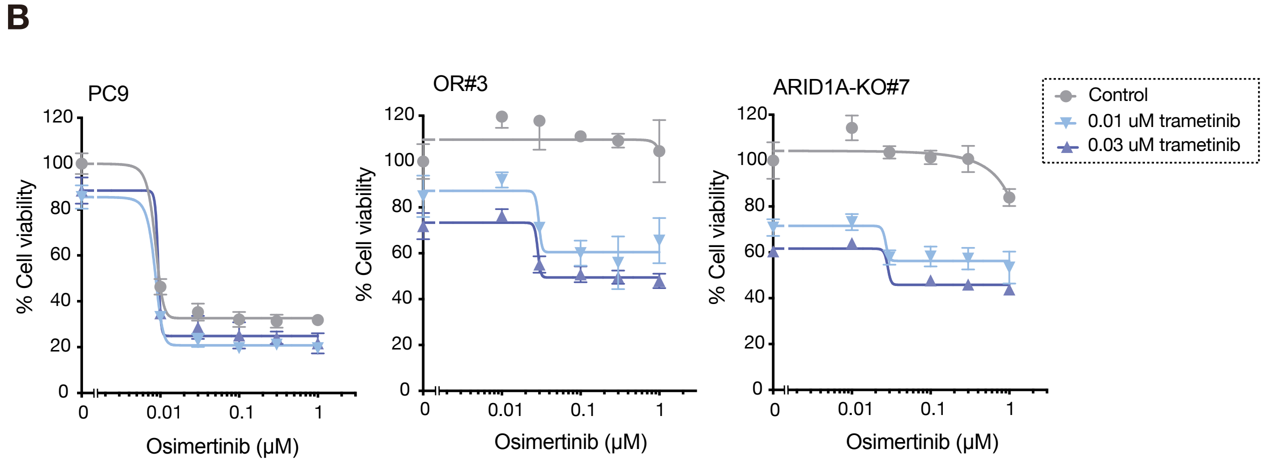

Figure S3 (A) PC9 cells and the resistant clones were treated with 1 μM osimertinib for 2 h. Changes in the phosphorylation levels of the indicated proteins were assessed by Western blotting. (B) PC9, OR#3 and ARID1A-KO#7 cells were treated with osimertinib in combination with trametinib. Cell viability was assessed using an MTT assay at 72 h. Bars represent mean ± SD of triplicate. Panel A adapted with permission from Cancer Science, 2021;112:3784-3795, Wiley.

Figure S4

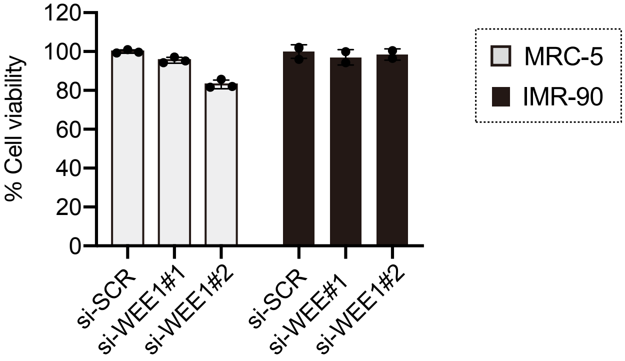

Figure S4 MRC-5 and IMR-90 cells were transfected with siRNAs targeting WEE1. Cell viability was assessed using an MTT assay at 72 h. Bars represent mean ± SD of triplicate.

Figure S5

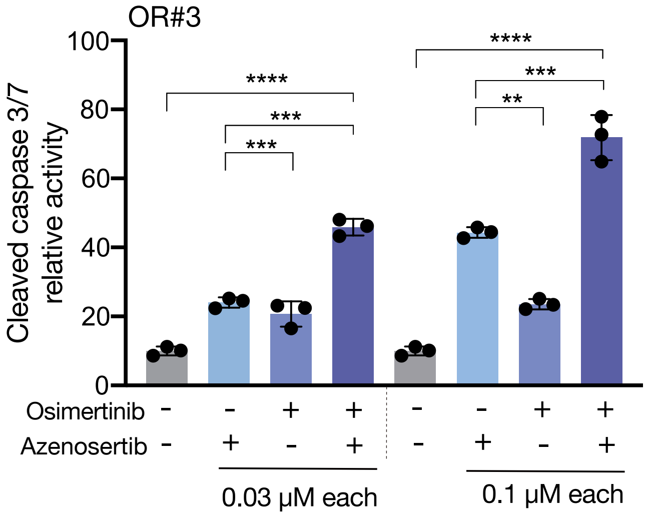

Figure S5 OR#3 cells were treated with osimertinib and/or azenosertib for 48 h. Apoptosis was quantified using the Caspase-Glo 3/7 assay. Bars represent mean ± SD of triplicate. Statistical significance was determined using Student's test. **p < 0.01, ***p < 0.001, ****p < 0.0001.

Table S1

Table S1. Summary of *EGFR* and *ARID1A* mutations in patients, related to Figure 6

Patient	*EGFR*	*EGFR*-T790M	*ARID1A*	*TP53*	*ARID1A* detection	CNS metastasis	PFS
TH-007	L858R	T790M	R1262C		before treatment	No	40
TH-067	Exon19del		F1859F	E286K. Q100*	before treatment	Yes	1
TH-107	Exon19del		A45-A46 insA	R158H	after treatment	Yes	2
TH-123	Exon19del	T790M	P490L		before treatment	Yes	4.5
TH-140	Exon19del	T790M	E59K	V272M	before treatment	Yes	1
TH-234	Exon19del	T790M	P508S		after treatment	No	1
TH-239	G719S E709A		S769*	P153fs	before treatment	Yes	1
TH-273	Exon19del		N935S	I251fs	after treatment	Yes	8
TH-301	L858R, Amp		D1963V, L2253L	G154V	after treatment	No	10
TH-329	Exon19del		A615V	TP53 splice site 920-1_920GC>TT	before treatment	No	19
TH-369	Exon19del	T790M	V1882M	C176R, Q331*	before treatment	No	27
TH-387	L858R		Q1573*	L330R	before treatment	Yes	10

Table S2

Table S2. Summary of lines of osimertinib treatment

ARID1A	1st line	2nd line or later
Mutant	2	10
Wild type	17	81

Fisher's exact test $p = 1.00$

Supplemental data

Materials and methods

Antibodies

Primary antibodies used in this study included EGFR (#4267; RRID: AB_2246311), ARID1A (#12354; RRID:AB_2637010), WEE1 (#13084; RRID: AB_2713924), phospho-CDK1 (Tyr15, #4539; RRID: AB_560953), ATM (#2873; RRID: AB_2052569), phospho-ERK1/2 (Thr202/Tyr204, #4370; RRID: AB_2315112), ERK1/2 (#4695; RRID: AB_390779), phospho-AKT (Thr202/Tyr204, #4370), AKT (#9272), cleaved caspase-3 (#9664; RRID: AB_2070042), γ-H2AX (#2577; RRID: AB_2118010), BRCA1 (#14823), BRCA2 (#10741), and β-actin (#4970; RRID: AB_2223172), all purchased from Cell Signaling Technology and used at a 1:1,000 dilution. Phospho-ATM (#ab81292; RRID: AB_1640207) was obtained from Abcam.

Western blotting

Cells were washed with PBS (Gibco) and lysed on ice using RIPA buffer (Thermo Fisher Scientific) supplemented with protease and phosphatase inhibitor cocktails (P8340 and P0044; Sigma-Aldrich, St. Louis, MO, USA). The lysates were collected, and equal amounts of protein (20 µg) were separated by electrophoresis on polyacrylamide gels (Mini-PROTEAN® TGX™ Precast Gels; Bio-Rad, Hercules, CA, USA) and transferred onto polyvinylidene difluoride (PVDF) membranes (Immun-Blot® PVDF Membrane; Bio-Rad). The membranes were blocked for 1 h

at room temperature with StartingBlock™ T20 (TBS) Blocking Buffer (Thermo Fisher Scientific), followed by overnight incubation at 4°C with primary antibodies (1:1000 dilution). Subsequently, the membranes were incubated with horseradish peroxidase (HRP)-conjugated secondary antibodies (#7074; Cell Signaling Technology) at a 1:2000 dilution for 1 h at room temperature. All antibodies were diluted in 5% (w/v) bovine serum albumin (BSA; Sigma-Aldrich) prepared in Tris-buffered saline (TBS) with 0.1% (v/v) TWEEN® 20 (TBS-T; Sigma-Aldrich). Between each step, the membranes were washed thrice with TBS-T for 10 min per wash. Immunoreactive bands were detected using SuperSignal™ West Dura Extended Duration Substrate (Thermo Fisher Scientific), and chemiluminescent signals were captured using a FUSION-SOLO Chemiluminescence Imaging System (Vilber Lourmat, Marne-la-Vallée, France).

Cell-viability assay

Cells were seeded at a density of 4,000 cells/well in 96-well plates and incubated overnight to allow attachment. Subsequently, the cells were treated with the specified compounds for 72 h. Cell viability was assessed using the MTT assay (Sigma-Aldrich), and absorbance was measured with an iMark™ Microplate Absorbance Reader (Bio-Rad). The percentage of cell viability was calculated relative to untreated control cells or baseline values. Half-maximal inhibitory concentration (IC_{50}) values were determined using a nonlinear regression model with a sigmoidal dose-response curve in GraphPad Prism 8 (GraphPad Software, La Jolla, CA, USA). Drug combination effects were analyzed using SynergyFinder

(https://synergyfinder.org/).

Apoptosis assay

Cells were seeded at a density of 4,000 cells/well in 96-well plates and incubated overnight to allow for attachment. Following incubation, the cells were treated with the specified compounds for 72 h. Apoptosis was assessed using the Caspase-Glo® 3/7 Assay (Promega, Madison, WI, USA) according to the manufacturer's protocol. Luminescence was measured using a Fluoroskan Ascent™ FL Microplate Fluorometer and Luminometer (Thermo Fisher Scientific). Simultaneously, cell viability was evaluated using the CellTiter-Glo® 2.0 Cell Viability Assay (Promega). Caspase 3/7 activity levels were normalized to cell viability to account for variations in cell number.

Cell cycle assay

Cells were seeded in 6-well plates at 30–50% confluence and treated with the specified agents the next day. After the designated incubation periods, cells were harvested and stained with Cell Cycle Assay Solution Deep Blue (Dojindo) for 15 min at room temperature. The stained samples were then analyzed by flow cytometry using an SH800 cell sorter (Sony).

Immunofluorescence staining

Cells cultured on chamber slides were fixed with ice-cold 100% methanol (FUJIFILM Wako) for 10 min at –20°C. After fixation, the cells were permeabilized

using 0.25% (v/v) Triton X-100 (Sigma-Aldrich) diluted in PBS for 10 min, followed by blocking with 5% (w/v) BSA in PBS for 30 min at room temperature. The cells were then incubated overnight at 4°C with primary antibodies diluted 1:100 in 5% (w/v) BSA/PBS. This was followed by a 1-hour incubation at room temperature with Alexa Fluor 488-conjugated secondary antibodies (#4412, 1:1000; Cell Signaling Technology). Finally, the nuclei were counterstained with DAPI (4′,6-diamidino-2-phenylindole) using VECTASHIELD® Antifade Mounting Medium with DAPI (Vector Laboratories, Burlingame, CA, USA). Mitotic catastrophe was visualized using a Leica TCS SP8 MP confocal microscope (Leica Microsystems), while γ-H2AX activity was assessed with an ECLIPSE Ti2 fluorescence microscope (Nikon).

siRNA transfections

Silencer® Select siRNAs targeting WEE1 (s21, s22), ATM (s1708, s1709), BRCA1 (s457, s458), BRCA2 (s2085, s224694), and RAD51 (s11734, s11735), along with *Silencer*® Select Negative Control siRNA #1 (#4390843), were obtained from Thermo Fisher Scientific. Cells were transfected with siRNAs using reverse transfection, performed with Lipofectamine™ RNAiMAX Transfection Reagent (Invitrogen, Waltham, MA, USA) following the manufacturer's protocol. Gene knockdown efficiency was validated via western blotting.

Generation of cDNA-expressing cell lines

The pcDNA6-ARID1A (#557328) and control pcDNA6 vectors were obtained from

Addgene. Cells were transfected using Lipofectamine™ LTX Reagent with PLUS™ Reagent, following the manufacturer's protocol. Successful gene expression was verified through western blot analysis.

CRISPR-Cas9 gene editing

Cas9-expressing cells were generated by transducing cells with lentiviral particles expressing Cas9 nuclease (LentiV_Cas9_puro; #108100; Addgene). For CRISPR knockout (CRISPR-KO) screening, synthetic single-guide RNA (sgRNA) libraries (Dharmacon Edit-R™; Horizon Discovery, Waterbeach, UK) were used following the manufacturer's protocol.

Mouse LMC model

To establish a leptomeningeal metastasis model, the scalp was sterilized with 70% ethanol, and cultured tumor cells (PC9-OR#3 harboring the *ARID1A* mutation; 4×10^5 cells in 0.1 mL) were injected into the leptomeningeal space between the external occipital protuberance and the first cervical vertebra using a 27-gauge needle. Tumor burden in live mice was monitored using noninvasive optical imaging via a tumor-specific luciferase assay and the IVIS Lumina XR imaging system (PerkinElmer). Ten minutes prior to imaging, the luciferase substrate (luciferin, 150 mg/kg) was administered intraperitoneally. Images were captured under bright-field illumination, and luminescence data were overlaid on the images. Luminescence signals were recorded within 1 to 30 s after anesthetizing the mice with 2% isoflurane. Bioluminescence signal intensity was

quantified as described previously (14).

After 2 weeks of treatment with osimertinib (25 mg/kg), the mice were randomly assigned to three groups and treated for 3 weeks with: (1) osimertinib alone (25 mg/kg/day), (2) ZN-c3 alone (60 mg/kg/day), or (3) a combination of osimertinib and ZN-c3. All treatments were administered daily via oral gavage. Osimertinib was dissolved in 0.5% methylcellulose and 1% Tween 80, while ZN-c3 was dissolved in a solution of 5% DMSO, 40% PEG 400, and 5% Tween 80. All animal experiments in this study were performed in strict accordance with the recommendations in the Guide for the Care and Use of Laboratory Animals of the Ministry of Education, Culture, Sports, Science, and Technology, Japan. The study protocol was approved by the Ethics Committee on the Use of Laboratory Animals and Advanced Science Research Center at Kanazawa University, Kanazawa, Japan (approval no AP-173867).

Statistical analysis

Data from the cell viability, apoptosis, and micronuclei assays were presented as means ± standard deviation (SD), while tumor progression data from animal studies were expressed as means ± standard error (SE). Kaplan-Meier curves were compared using the log-rank test.

Statistical analyses were performed using GraphPad Prism (Version 8.0). A *p*-value of less than 0.05 was considered statistically significant, with significance levels denoted as follows: ns (not significant) > 0.05, $^{*}p < 0.05$, $^{**}p < 0.01$, $^{***}p < 0.001$, and $^{****}p < 0.0001$.

Human EGFR TKI–treated deidentified patient cohort

Institutional Review Board (IRB) approval for study no. 13-12492 was obtained from the UCSF IRB. In accordance with federal regulations outlined in 45 CFR 46.102(f), this study did not involve human subjects and, therefore, did not require additional IRB oversight. The requirement for informed consent was waived. The study investigators conducted a retrospective chart review to collect deidentified patient demographic data, including the incidence of CNS metastases, progression-free survival (PFS), and overall survival (OS) following EGFR-TKI therapy. Both PFS and OS were measured from the data of osimertinib initiation rather than from diagnosis or biopsy date.
